# Efficient Signed Certificate Verification for IoT and V2V Messages via Blockchain Integration

**DOI:** 10.3390/s25247528

**Published:** 2025-12-11

**Authors:** David Khoury, Khouloud Eledlebi, Kassem Hamze, Jinane Sayah, Patrick Sondi, Kassem Danach, David Semaan, Hassan Farran, Samir Haddad

**Affiliations:** 1 Laboratoire d’Informatique Signal et Image de la Côte d’Opale (LISIC UR 4491), Université du Littoral Côte d’Opale, 59375 Calais, France; dkhoury@aust.edu.lb; 2 Department of Computer Science and IT, College of Engineering, Abu Dhabi University, Abu Dhabi P.O. Box 59911, United Arab Emirates; khouloud.eledlebi@adu.ac.ae; 3 Faculty of Engineering, Islamic University of Lebanon, Beirut P.O. Box 30014, Lebanon; kassem.hamze@iul.edu.lb; 4 Department of Telecom and Networks, Issam Fares Faculty of Technology, University of Balamand, Koura P.O. Box 100, Lebanon; 5 Center for Digital Systems, IMT Nord Europe, Institut Mines-Télécom, 59650 Lille, France; patrick.sondi@imt-nord-europe.fr; 6 Basic and Applied Sciences Research Center, Al Maaref University, Beirut P.O. Box 5078/25, Lebanon; kassem.danach@mu.edu.lb; 7 Faculty of Engineering and Computer Science, American University of Science and Technology (AUST), Beirut P.O. Box 16-6452, Lebanon; dsemaan@aust.edu.lb; 8 Department of Networked Systems and Services, Faculty of Electric Engineering and Informatics, Budapest University of Technology and Economics, 1111 Budapest, Hungary; hassanfarran@edu.bme.hu; 9 Department of Computer Science and Mathematics, Faculty of Arts and Sciences, University of Balamand, Koura P.O. Box 100, Lebanon; samir.haddad@balamand.edu.lb

**Keywords:** Internet of Things (IoT), Vehicle-to-Everything (V2X), certificates, public key infrastructure (PKI), certificate authority (CA), TLS/DTLS, blockchain

## Abstract

Symmetric cryptographic schemes such as RSA and ECDSA (Elliptic Curve Digital Signature Algorithm), used for digital signatures in protocols like TLS, DTLS, and secure messaging, are computationally intensive. This makes them unsuitable for constrained environments, such as the Internet of Things (IoT) and the Internet of Vehicles (IoV). This study introduces a blockchain-based framework that utilizes the Ethereum network to store and verify public keys associated with digital certificates. By replacing signature decryption with blockchain-based public key verification, the solution significantly reduces cryptographic overhead and latency in V2V messages. It supports various certificate formats, including Public Key Infrastructure (PKI)/Certificate Authority (CA) certificates such as X.509 and L-ECQV, as well as self-signed certificates. Applications include secure communication protocols like Datagram Transport Layer Security (DTLS)/Transport Layer Security (TLS), V2V mutual authentication in V2X messaging, and lightweight certificate management within IoT ecosystems. Empirical results show that the DTLS handshake with this scheme is reduced from 12 s to less than 6 s. Additionally, it enables vehicles and IoT devices to perform one-time signature verification with minimal latency in V2V messaging, demonstrating significant performance improvements for high-density deployments involving mutual authentication between IoT devices and V2V communication.

## 1. Introduction

Traditional authentication mechanisms for digital communications, such as those based on the X.509 standard [1], rely heavily on Public Key Infrastructure (PKI) and Certificate Authorities (CAs). These mechanisms establish a hierarchical trust model wherein CAs issue digital certificates to authorized entities, binding public keys to verified identities. The certificates form part of a “chain of trust,” enabling secure communication channels through protocols such as Transport Layer Security (TLS) or Datagram Transport Layer Security (DTLS) [2,3].

Although robust, these X.509 certificates present limitations when applied to the Internet of Things (IoT) and the Internet of Vehicles (IoV) domains. Specifically, they are computationally intensive and often have a large footprint, unsuitable for resource-constrained devices. The verification process, which includes cryptographic signature validation and, sometimes, certificate chain handling, imposes significant latency and energy consumption due to asymmetric decryption barriers that limit the capabilities of real-time or high-density devices.

Cooperative Intelligent Transport Systems (C-ITS) rely on dependable Vehicle-to-Everything (V2X) communication, enabling seamless data sharing among vehicles, infrastructure, pedestrians, and network services. This extensive communication framework encompasses Vehicle-to-Vehicle (V2V), Vehicle-to-Infrastructure (V2I), Vehicle-to-Network (V2N), and Vehicle-to-Pedestrian (V2P) exchanges, facilitating key functions such as collision avoidance, traffic control, and autonomous driving. Its primary aims are to enhance road safety, boost efficiency, and support environmental sustainability [4].

Current C-ITS security frameworks primarily rely on the Vehicle Public Key Infrastructure (VPKI), as defined by ETSI in Europe [5]. This involves a hierarchy of Certificates by Enrolment Authorities (EAs) and Authorization Authorities (AAs), with vehicles carrying long-term IDs and short-term Authorization Tickets (ATs) for message signing. Nonetheless, this centralized setup, VPKI, encounters issues, mainly due to the size of the certificate and the intensive processing required to verify message signatures, leading to noticeable delays [6].

To address these constraints, researchers have explored alternative, lightweight certificate designs that are compatible with PKI/CA frameworks. Among the most notable are:

- L-ECQV (Lightweight Elliptic Curve Qu-Vanstone) implicit certificates [7,8],
- L-ECQV explicit certificates [9],
- Concise Binary Object Representation (CBOR) binary-compressed lightweight X.509 certificates [10].

In parallel, blockchain-based certificate schemes such as LightCert4IoT have emerged [11]. These certificates are self-signed, compact, and stored immutably on blockchain networks such as Ethereum. By decentralizing certificate issuance and verification, they remove the need for trusted third-party authorities and improve automation.

Even with lightweight certificates introduced, verifying signed certificates remains challenging because decryption is required, which introduces significant latency—a critical issue in V2V messaging. To address this issue, this research paper proposes a blockchain-based certificate verification solution, similar to the DTLS handshake signature verification described in [12]. The main contribution of this document is the following:

(1) Expand the DTLS solution to support many secure communication protocols, especially during the handshake phase, where entities exchange digital certificates to establish mutual trust. This approach applies to protocols such as TLS and DLS, as well as other secure channel mechanisms between clients and servers that depend on this process to authenticate identities and establish encrypted sessions, including IKEv2 and custom mutual authentication schemes. Verification of the certificate on both the IoT device and the server, removing the need for traditional methods.
(2) Further reduction of latency in V2V communication by leveraging blockchain technology. It proposes replacing the decryption of the AT certificate signature with verification of the pre-stored AT and AA public keys on the Distributed Public Key (DPK) blockchain platform [13]. This approach greatly reduces cryptographic overhead in V2V mutual authentication for V2X communications.

This study proposes a novel, global approach that integrates certificate verification with blockchain technology, leveraging Ethereum’s decentralized infrastructure via the DPK platform. The use of Ethereum, a permissionless, globally maintained blockchain, serves as a proof-of-concept for the proposed framework. While computationally intensive, Ethereum’s transparency and maturity enable rapid prototyping. In practical deployments, the system can be adapted to operate on a private blockchain, leveraging optimized consensus mechanisms to enhance throughput and reduce resource costs. The detailed contributions of this work are summarized as follows:

1. A universal mechanism for storing and signature verification for all certificate types, not limited to X.509, L-ECQV (implicit and explicit), LightCert4IoT, and certificate digests—using the DPK Ethereum blockchain.
2. A DPK blockchain-based public key verification method that eliminates the need for decrypting digital signatures during authentication, thereby minimizing cryptographic load and reducing latency in V2V messaging. This process replaces decrypting the AT certificate signature with verifying the AT public key that is pre-stored in the DPK blockchain.
3. Pre-stored public keys in DPK of the pseudonym certificates (ATs) are utilized to avoid the computational time required to reconstruct the AT’s actual public key during V2V message signature verification, eliminating the need to combine the AT device’s public key reconstruction data from the certificate with the certificate authority’s public key.
4. A method for storing CA or AA (in case of V2X) public keys on the blockchain, enabling lightweight reconstruction by IoT and V2X receivers based on CA or AA identifiers.
5. Application of the proposed method in Vehicle-to-Vehicle (V2V) communication, improving latency and scalability for Cooperative Awareness Messages (CAMs) and Decentralized Environmental Notification Messages (DENMs).

In summary, the novelty of this paper is a universal system for storing certificates and public keys on the DPK Ethereum blockchain. It includes a public-key verification method that eliminates the need to decrypt digital signatures during authentication, thereby reducing the cryptographic workload and decreasing latency in V2V messaging.

This research paper does not aim to make a quantitative comparison with other blockchain PKI systems. Still, it allows us to demonstrate the feasibility of an efficient signed certificate verification method for IoT and V2V messages via Blockchain Integration. We adopted Ethereum, a permissionless blockchain, as a proof-of-concept for our implementation. The advantage of Ethereum is that it is open-source and maintained by a global consortium, a continuously evolving network.

The remainder of this paper is organized as follows: Section 2 reviews foundational technologies and provides an overview of the DPK platform and prior work; Section 3 presents the architecture of the proposed blockchain-based certificate verification system; Section 4 explores certificate verification messages in V2X. Section 5 outlines experimental evaluations and performance benchmarks; Section 6 concludes with key findings and future directions; and Section 7 lists related work.

## 2. Background

### 2.1. Overview of DPK Platform

The Distributed Public Key (DPK) platform is a blockchain-based infrastructure designed to facilitate decentralized storage and retrieval of cryptographic credentials, including certificates and public keys. Built on the Ethereum network, the DPK framework enables secure, verifiable identity management without dependence on centralized Certificate Authorities (CAs). The underlying architecture is described in [14] and further supported by the U.S. Patent [13]. The core components of the DPK platform include:

#### 2.1.1. Wallet Management Module (PKM)

The Public Key Manager (PKM) is a network function on the server side of the DPK platform. It authenticates DPK module users and approves Blockchain storage requests. It contains Wallet Management (WM) functions that transfer Ether to the DPK client module after the device’s identity is confirmed and its transaction fees are paid through the associated Token. The PKM contains the application provider function that executes transactions directly on the blockchain, similar to INFURA.

The primary feature of the PKM is wallet management. When users download or install the DPK client-side module, it automatically generates Ethereum public and private keys, creating an empty Ethereum wallet. The Wallet Management module primarily authenticates the DPK client module on the device and facilitates the transfer of essential Ether to it. This Ether allows the client application to store user data transactions on the Ethereum network.

#### 2.1.2. Local Registration Authority (LRA) Nodes

Acting as trusted intermediaries, LRA servers validate application-specific identities, such as those of IoT devices, and interact with the blockchain to store or update certificate data. LRAs are also responsible for deploying smart contracts relevant to device applications. In specific configurations, LRAs function as full Ethereum nodes, capable of mining, validating, and maintaining decentralized ledger entries.

#### 2.1.3. Client-Side DPK Module

This lightweight software plug-in is installed directly on user equipment (e.g., mobile phones, IoT sensors, V2X devices). Upon initialization, it creates a unique Ethereum address for each device, manages user-generated public keys, and coordinates secure data uploads to the blockchain via smart contract interactions. Its essential features are:

- The DPK module operates on user equipment to manage user public keys securely.
- It interacts with a Public Key Manager (PKM) to obtain cryptocurrency for transactions.
- User public keys are generated by applications and stored in the blockchain, enhancing security.

#### 2.1.4. Smart Contract

Deployed on the Ethereum blockchain, smart contracts govern the secure storage, retrieval, and verification of user credentials. These contracts ensure that only authenticated and authorized actions are executed, protecting the integrity and availability of the credential database. To enable interaction between user devices and the blockchain, a universal DPK client module is embedded into the device firmware or installed as a secure plug-in. This module communicates with PKM to perform mutual authentication and acquire the necessary cryptocurrency tokens to pay for blockchain transactions (e.g., gas fees).

The process of storing the public key is outlined as follows:

1. The user’s public key is initially generated locally on the device, typically via an application (APP) running on the user equipment.
2. The public key is then transmitted to the DPK module, which verifies its authenticity and constructs a storage transaction request.
3. Upon approval, the DPK module transmits the user’s public key to the blockchain using a smart contract, which governs the interaction protocol between the DPK module and the blockchain ledger.
4. The public key is then stored securely within a transparent Ethereum address associated with the device, ensuring traceability and tamper-resistance.

Importantly, the user’s public keys are never stored off-chain or on third-party servers, eliminating external attacks and reducing the risk of key compromise.

The features associated with the DPK Platform are the following:

- **Decentralized Trust Model**: The platform replaces traditional centralized PKI with a decentralized blockchain framework, removing the single point of failure associated with Certificate Authorities.
- **End-to-End Key Integrity**: Only verified and authorized transactions can update or register public keys, ensuring strong guarantees of data integrity and authenticity.
- **Enhanced Privacy and Security**: As public keys reside solely on the blockchain and are never replicated off-chain, the risk of unauthorized access or key leakage is significantly mitigated.
- **Smart Contract Automation**: The use of smart contracts automates and enforces the logic of public key registration, updates, and verifications, enabling trustless operations without intermediary services.
- **Device-Level Integration**: The DPK module integrates seamlessly with IoT devices, allowing real-time secure communications, certificate updates, and blockchain interaction with minimal overhead

### 2.2. Lightweight ECQV for Authentication on the Internet of Things

Traditional X.509 certificates [1], while standardized and widely supported, pose significant limitations in Internet of Things (IoT) environments due to their large size and processing overhead. The complexity of X.509 structures, along with the associated metadata and signature chains, makes them inefficient for constrained devices with limited computational power, memory, and bandwidth. As a more suitable alternative, Elliptic Curve Qu-Vanstone (ECQV) certificates, particularly in their lightweight (L-ECQV) variant, offer a compact, computationally efficient solution for establishing secure, authenticated communications in IoT networks [7,8]. L-ECQV certificates require substantially less storage space and offer faster processing times than X.509 certificates, making them better aligned with the resource constraints of IoT systems.

However, a key challenge in public key infrastructure is certificate revocation. Mechanisms such as Certificate Revocation Lists (CRLs) and the Online Certificate Status Protocol (OCSP), typically issued by centralized Certificate Authorities (CAs), involve frequent network communication and consume significant memory and bandwidth. These requirements make revocation management particularly difficult in decentralized or multi-CA IoT deployments [15].

To further optimize performance, the L-ECQV certificate format can be encoded using Concise Binary Object Representation (CBOR). This lightweight binary serialization format further reduces the certificate size without compromising structure or semantics. This combination of elliptic curve cryptography and compact encoding techniques represents a significant advancement in secure identity management for IoT.

L-ECQV certificates can be categorized into two forms: explicit, in which the public key is included in the certificate, and implicit, in which the public key is reconstructed by the verifier using auxiliary data and the CA’s public key [9]. Both forms support cryptographic unlinkability and enhanced privacy, making them highly applicable to dynamic, large-scale IoT networks.

The value of ***p*** depends on the elliptic curve used and corresponds to the byte length of an EC point. Table 1, based on [16], provides the exact sizes of commonly used curves, such as NIST P-256 and P-384. By excluding the CA’s signature and leveraging on-the-fly public key reconstruction, the L-ECQV implicit scheme offers a lightweight, secure, and efficient alternative to traditional certificate formats, particularly suitable for bandwidth-constrained and computationally limited IoT environments.

L-ECQV Implicit Certificates (see Table 2). The tables are extracted from reference [16].

The L-ECQV implicit certificate scheme is designed to reduce the certificate overhead in constrained IoT systems. In this mechanism, the certificate authority’s (CA) digital signature is omitted from the certificate issued to the IoT device. Instead, the certificate contains cryptographic reconstruction data that enables a verifier to dynamically derive the sender’s public key.

During communication, when an IoT device transmits a signed message, the receiving device retrieves the reconstruction data embedded in the certificate. It combines it with the CA’s public key to reconstruct the sender’s public key. This reconstructed key is then used to verify the digital signature on the received message, ensuring authenticity and integrity without requiring the storage or transmission of a complete public key or certificate chain.

The sizes of its components determine the length of the L-ECQV implicit certificate. Let **p** denote the length of the elliptic curve public key (typically represented as an EC point). In addition to the public key reconstruction data, the certificate includes metadata fields such as Certificate type, Serial number, Curve identifier, Hash algorithm, Issuer identifier, Validity period (valid from, valid to), Subject identifier, and Key usage permissions. The total length of these metadata fields is **37 bytes**, as specified in Table 2. Therefore, the overall size of an L-ECQV implicit certificate is calculated as:

(1) Certificate Length=37+p bytes

L-ECQV Explicit certificate builds on the structure of the lightweight implicit certificate, with a critical distinction: the public key field contains the IoT device’s actual elliptic-curve public key, rather than reconstruction data. Additionally, the certificate includes a digital signature generated by the CA, thereby enabling immediate public key validation without requiring the reconstruction of the key. This explicit inclusion of both the public key and the CA’s signature increases the overall certificate size. Still, it simplifies the verification process, as the receiver does not need to reconstruct the public key from auxiliary data. The total length of the lightweight explicit certificate can be expressed as:

(2) Certificate Length=37+p+s bytes

where **s** represents the byte length of the CA’s ECDSA signature over the certificate. The values of ***p*** and ***s*** vary based on the elliptic curve parameters and whether compressed or uncompressed EC point formats are used, as in Table 3. For instance, using NIST P-256 with a compressed EC point, the typical values are:

- ***p* = 33 bytes** (compressed public key),
- ***s* = 65 bytes** (ECDSA signature),

Yielding a total certificate size of:

(3) Total Length=37+33+65=135 bytes

The inclusion of the CA signature enhances trust and verifiability at the cost of a modest increase in size, making the lightweight explicit certificate a practical option when immediate and standalone public key verification is desired without reliance on external public key reconstruction mechanisms. This format is particularly suitable in IoT deployments where some devices have limited computation power but sufficient memory to accommodate slightly larger certificate sizes. L-ECQV Explicit Certificates (see Table 4).

### 2.3. Certificates Digest

The certificate digest, introduced in paper [16], aims to minimize transmission and verification costs in environments with frequent or repetitive message exchanges. It involves the cryptographic hash (e.g., SHA-256) of the entire lightweight certificate, whether implicit or explicit L-ECQV. This method is particularly effective when a device transmits a series of (m) packets within a specific period. The initial packet carries the full certificate, while subsequent packets only include the digest, significantly reducing redundancy and bandwidth usage.

The digest typically uses the HashedID_H format, where H represents the digest length. A common choice is H = 8 bytes, consistent with standards such as IEEE 1609.2 [17], which specify 8-byte fields for certificate identifiers, including the issuer ID and serial number. Using a smaller H might increase hash collisions, while a larger H could result in higher storage requirements. This method has been tested on hardware like the Raspberry Pi 4 Model B, demonstrating its ability to reduce cryptographic and communication overhead without compromising security.

### 2.4. LightCert4IoT

As IoT systems expand alongside the deployment of 5G and future 6G technologies, the need for scalable, low-overhead authentication mechanisms becomes increasingly pressing. Traditional PKI-based certificate issuance faces multiple challenges in dynamic and decentralized environments, including high costs, scalability bottlenecks, and inefficiencies in certificate revocation. To address these limitations, researchers have proposed LightCert4IoT, a lightweight, self-signed certificate framework specifically designed for constrained IoT devices [11]. Unlike traditional certificates issued by CAs, LightCert4IoT certificates are self-generated and self-signed by the end-user devices (e.g., mobile nodes, sensors, or embedded systems), thereby eliminating the cost, latency, and complexity associated with hierarchical certificate issuance.

LightCert4IoT is built on the DPK infrastructure of the Ethereum blockchain, where LRAs or Edge nodes are responsible for verifying and validating the binding between a user’s identity and their self-signed certificate. The Ethereum blockchain serves as a global, decentralized notary, providing an immutable ledger for securely storing IoT certificates. This architecture ensures tamper-resistant, transparent, and verifiable public key management without relying on centralized trust anchors. By integrating smart contracts, LightCert4IoT enables secure, automated processes for certificate issuance, updates, and revocation. These operations are executed in a decentralized manner, ensuring trustless interoperability and improved fault tolerance. Additionally, LightCert4IoT significantly reduces cryptographic and communication overhead, making it well-suited for low-power, memory-constrained IoT devices.

#### Certificate Revocation

When traditional X.509 certificates are issued to domains, they are expected to remain valid throughout their specified period. However, certificates are revoked if they become untrustworthy, such as when they pass their expiration date, or peers deem them invalid and untrusted. Another main reason for revocation is the compromise of encryption keys. In DPK, revoking a certificate involves replacing it with a new one. When a V2X or IoT certificate is stored in DPK Ethereum, revocation is managed by the IoT device updating its record in the Ethereum blockchain smart contract, with approval from the LRA. Each smart contract record includes the device’s wallet address. To revoke a certificate, the device updates its corresponding record in the contract. The process begins when a client is prevented from initiating an update request for the wallet used to create its identity without the LRA’s approval.

Experimental evaluations of the LightCert4IoT system demonstrate that it achieves better performance metrics than existing PKI-based solutions. The results confirm its suitability for real-world IoT deployments, offering a scalable and secure alternative to traditional certificate management systems in decentralized environments [18].

### 2.5. Vehicle PKI in V2X, Long-Term and Pseudonym Certificates

The security infrastructure for V2X communication primarily relies on Public Key Infrastructure (PKI). Within the European framework, the ETSI C-ITS Trust Model establishes a hierarchical certificate structure that includes Root Certificate Authorities (CAs), Enrollment Authorities (EAs), and Authorization Authorities (AAs). Vehicles are provisioned with long-term enrollment certificates, known as EC or EA, for identity management, and short-term pseudonym certificates, known as Authorization Tickets (ATs), to ensure user privacy, created by the AA. [19]. Outgoing messages in V2V are digitally signed using the vehicle’s AT private key, with the corresponding Authorization Ticket, containing the vehicle’s public key, appended to the message. Message recipients must then validate the message’s authenticity by verifying the signature against the issuing Authorization Authority’s public key [20].

The AT certificate is a type of implicit L-ECQV designed to reduce the certificate overhead. The certificate authority’s (AA) digital signature is omitted; instead, the certificate contains cryptographic reconstruction data that enables a verifier to derive the sender’s public key dynamically.

When a V2X device transmits a signed message, the receiving device retrieves the reconstruction data embedded in the certificate. It combines it with the AA’s public key to reconstruct the sender’s public key. This reconstructed key is then used to verify the digital signature on the received message, ensuring authenticity and integrity without the need to store or transmit a complete public key or certificate chain.

ITS users’ privacy is protected by regularly updating certificates and using pseudonym certificates to authenticate safety messages. This strategy makes vehicle tracking difficult, if not nearly unfeasible. To preserve privacy, the PKI must generate and supply multiple pseudonym certificates to each Intelligent Transport System Station (ITS-S). Vehicles frequently change their digital identities using pseudonym certificates, thereby enhancing driver privacy and making tracking more challenging. They can select from a pool of up to 60 pseudonyms each week for V2V communication, and up to 100 to meet European Commission standards. This set of pseudonyms needs to be updated regularly and replenished.

#### Explanation of Figure 1

**Enrollment Authority (EA):** This authority primarily verifies and grants permissions for ITS stations to engage in communications. Each ITS station is assigned a unique ID, along with cryptographic keys (both Public and Private) created during the initialization stage. Following this, the enrollment phase begins, during which the ITS-S verifies its identity with the EA to receive enrollment credentials. These credentials allow access to particular ITS applications and services. Only the EA holds the actual identity of an ITS-S. The EA issues enrollment credentials (EC), which serve as identification certificates. These credentials provide the device with long-term identification and confirm that it satisfies the necessary security standards for V2X communications.

**Figure 1:**
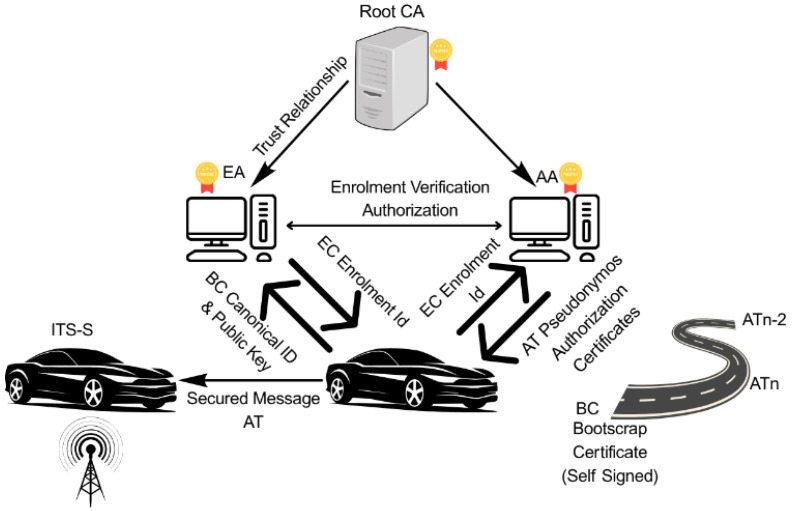
Architecture of C-ITS VPKI.

**Authorization Authority (AA):** Its primary function is to issue multiple Authorization Tickets (AT) to an ITS station after validating its Enrollment Credentials (EC) with the EA. Authorization Tickets are pseudonymous certificates that are frequently exchanged to protect the real identity of the ITS station. The AA issues certificates known as authorization tickets (AT). These tickets serve as pseudonymous certificates, helping to anonymize the entity and prevent traceability. Devices utilize ATs for V2X messages such as Cooperative Awareness Messages (CAM) [21], Decentralized Environmental Notification Messages (DENM) [22], and Basic Safety Messages (BSM).

The Common Awareness Messages (CAM) provide status information, and additionally, Decentralized Environmental Notification Messages (DENM) include alerts for road work. Cooperative Awareness Messages (CAMs) and Decentralized Environmental Notification Messages (DENMs) rely on real-time information sharing to enhance safety, streamline traffic management, and support the development of automated driving capabilities [20]. BSM is a standardized message defined in the US standards SAE J2735 and SAE J2945/1 [23,24], designed to support Vehicle-to-Vehicle (V2V) and Vehicle-to-Infrastructure (V2I) safety applications. It contains critical vehicle state information that helps nearby vehicles and roadside units (RSUs) make informed decisions.

The EA and AA are linked together. Once a V2X message is validated, an authorization validation request is submitted to the EA, which verifies the sender’s identity and permissions and returns an authorization validation response. For security reasons, the EA and AA are rigorously separated by physical location and personnel, preventing simultaneous access to the vehicle’s long- and short-term identity information. The main difference between PKI and V2X PKI lies in the continuous renewal of certificates, which is necessitated by the short-lived ATs (pseudo-certificates) in the Internet of Vehicles.

## 3. Certificate Verification-Based Blockchain

Traditional methods for verifying certificates assigned to IoT devices and servers during the handshake phase of secure communication protocols, such as Datagram Transport Layer Security (DTLS) [3] or Transport Layer Security (TLS) [2], suffer substantial computational and energy overhead. This is primarily due to the reliance on asymmetric cryptographic operations, including decryption and validation of digitally signed X.509 certificates. These processes substantially increase latency and energy use, especially in environments with limited resources. As mentioned in [12], empirical evaluations show that the cryptographic overhead per DTLS handshake can reach up to 15 s, even with minimal certificate chains. We proposed a method in the paper [12] to reduce handshake time in DTLS. We suggest a certificate verification approach that bypasses the need to decrypt or fully validate the signed certificate during session initialization. Instead, it confirms a certificate’s authenticity by directly referencing the certificate’s public key, which is stored offline on the DPK platform and runs on a blockchain. This enables quick, lightweight verification without the need for traditional signature decryption during the handshake. The method is protocol-agnostic, enabling seamless integration with various secure communication protocols, including TLS, DTLS, and custom protocols, without altering the core security architecture. We aim in this chapter to generalize the blockchain-based certificate verification method to different certificate types, whether issued by standard PKI and CAs, such as L-ECQV, or created through decentralized or self-signed methods, such as LightCert4IoT.

### 3.1. Pre-Storage of Certificates and Public Keys in the DPK Platform

**Figure 2:**
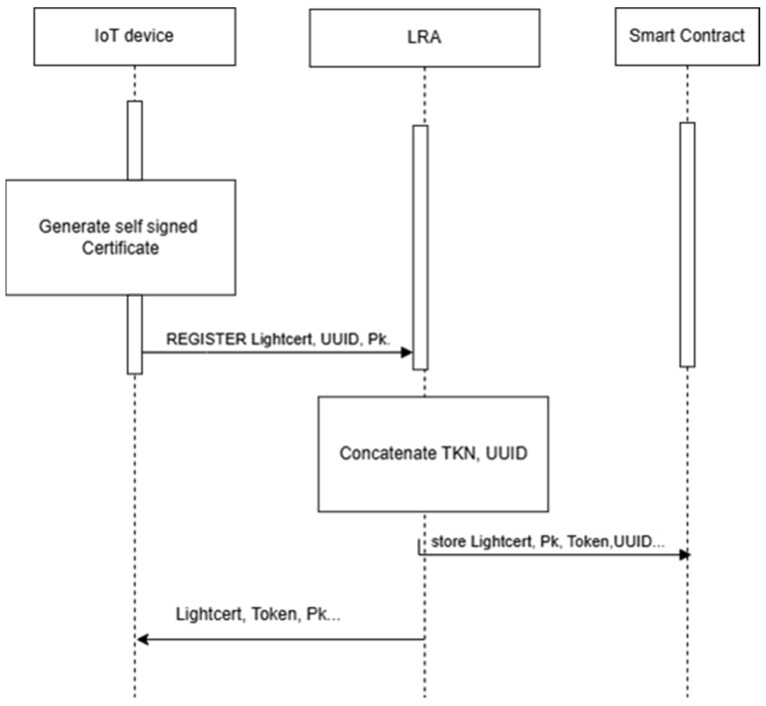
Storage of the self-signed certificate in DPK.

Certificates issued under PKI and signed by CAs can be securely stored within the DPK platform. This architecture offers several advantages, notably the ability to efficiently verify signed certificates, as demonstrated in [12]. Before storage, the certificate must undergo authentication, which traditionally involves verifying the CA’s digital signature by decrypting the certificate using the CA’s public key. Upon successful validation, the IoT device, via the LRA, initiates a secure transaction to store the certificate and its associated public key on the DPK blockchain. This process aligns with the approach outlined in the LightCert4IoT framework [11] and illustrated in the sequence diagram in Figure 2.

The proposed solution leverages the immutability and transparency of blockchain to securely pre-store the certificate data and its corresponding public key. During the handshake process, the recipient device retrieves the pre-stored public key from the DPK blockchain, ensuring that the certificate is valid and bound to the correct entity, without incurring the computational cost of real-time cryptographic validation. By offloading certificate verification to a blockchain-based lookup mechanism, this method not only reduces session establishment latency and energy consumption but also enhances scalability and performance in dense IoT environments.

**Figure 3:**
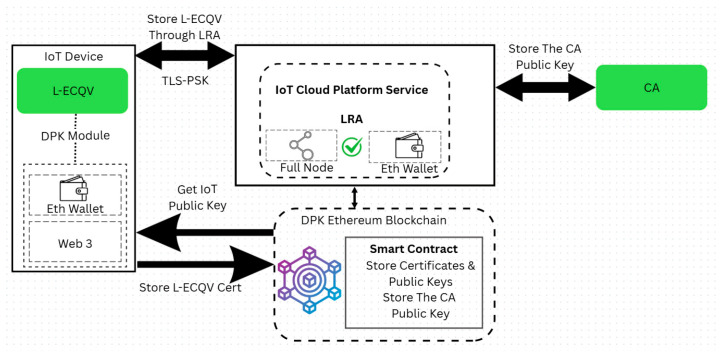
System Architecture: Storage of the certificate, its public key, and the CA public key in the DPK blockchain.

Figure 3 illustrates a system architecture for pre-storing the L-ECQV certificate, its public key, and the CA public key in the DPK blockchain.

### 3.2. Pre-Storage of the Case L-ECQV Certificates and Public Keys

For L-ECQV implicit certificates, the CA’s digital signature is excluded from the certificate payload to minimize overhead. Instead, the public key field contains cryptographic reconstruction data. During storage, this reconstruction data, along with other certificate metadata, is submitted to the blockchain. Once the public key is derived using the CA’s public key, both the reconstructed public key and the certificate content are securely pre-stored in the DPK. To enable public-key reconstruction for L-ECQV implicit certificates, the CA’s public key must also be stored in the DPK platform. This ensures that receiving entities can reliably reconstruct the sender’s public key during future communications. After authenticating the certificate, the IoT device, through the LRA, executes blockchain transactions to store both the user certificate and the CA’s public key on the Ethereum-based DPK ledger. The LRA server may also operate as a full Ethereum node, participating in blockchain operations such as transaction validation, smart contract execution, and, where applicable, consensus (e.g., mining or staking under different Ethereum protocols).

A smart contract deployed within the DPK platform acts as the certificate registrar, managing the validation, storage, and lookup of public keys and certificates. This contract enforces strict authentication logic and guarantees data immutability, tamper-resistance, and availability across the blockchain network. The integration of CA public keys in this manner enables secure, decentralized certificate management across various certificate formats, including both X.509 and L-ECQV.

### 3.3. Signature Certificate Verification During the Handshake of Secure Protocols

**Figure 4:**
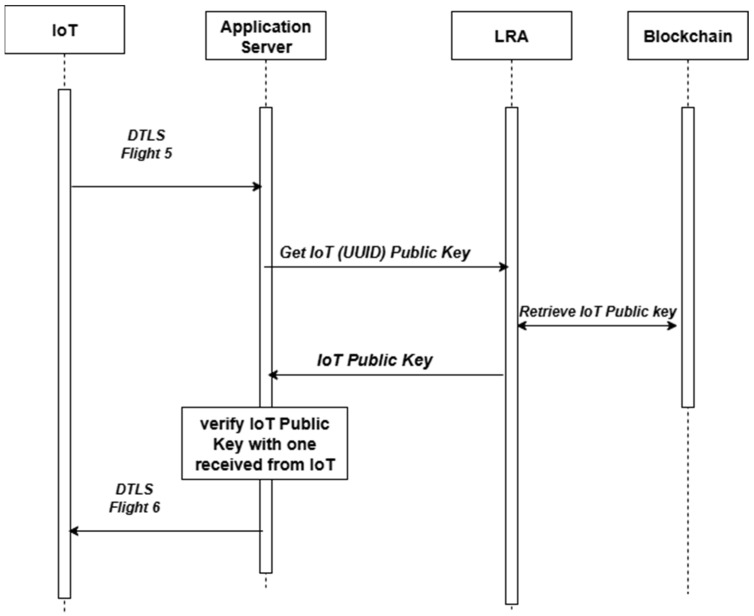
Certificate verification in the case of the DTLS protocol.

Signature verification, as described in [12], is a fundamental operation in secure communication protocols, particularly during the handshake phase, when entities exchange digital certificates to establish mutual trust. In this method, during the handshake, instead of decrypting and verifying the certificate signature using traditional PKI/CA methods, the recipient entity retrieves the public key from the blockchain. It compares it against the public key included in the received certificate. If the keys match, the certificate is considered valid and trusted. This technique has been specifically implemented and tested within the DTLS handshake protocol, as presented in [12]. Refer to Figure 4 for certificate verification in the DTLS protocol.

By offloading public key verification to the blockchain and avoiding real-time cryptographic signature validation, this method streamlines the handshake process, significantly reduces computational complexity, and lowers energy consumption. These advantages make it particularly well-suited for low-power and latency-sensitive applications in dense IoT environments or edge computing systems, where traditional PKI-based handshakes may be infeasible or too costly in terms of performance.

For self-signed certificates, such as LightCert4IoT, the entire certificate, along with its public key, is stored on the blockchain. During the handshake security protocol, the receiver device or server retrieves the matching public key through a blockchain query. It then compares it with the one received from the sending device; if they match, authenticity is confirmed.

For implicit L-ECQV certificates, the certificate contains reconstruction data but not the full public key (see Table 2). After reconstructing the certificate’s public key, it is stored and validated in the blockchain along with the CA’s public key. This mechanism can occur offline. Later, receiving devices can retrieve the certificate’s public key from the blockchain, which serves as a trusted ledger platform, and validate the certificate’s authenticity. This method eliminates the need for real-time signature decryption and can be applied uniformly across various certificate formats and communication protocols.

This modification significantly reduces processing time and energy consumption during the handshake by bypassing traditional certificate decryption and signature verification processes. This is especially advantageous in resource-constrained IoT environments, where performance, memory, and power efficiency are critical.

### 3.4. Mutual Authentication for IoT, V2V Devices

In Vehicle-to-Vehicle (V2V) and broader Internet of Things (IoT) communication scenarios, data exchange between devices, such as sensors, actuators, or connected vehicles, occurs independently of the underlying packet transmission protocol. To ensure secure, authenticated communication, each transmitted message is typically signed by the sender’s private key and accompanied by a digital certificate that verifies the identity of the sending device and its public key. However, this conventional approach introduces significant computational overhead due to its reliance on public-key cryptography for signature validation. Each certificate must be individually verified using the CA’s or AA’s (in case of V2V) public key, which increases latency and energy consumption, especially in high-frequency message streams typical of V2V environments.

#### 3.4.1. Certificate Public Key Pre-Stored in DPK

**Figure 5:**
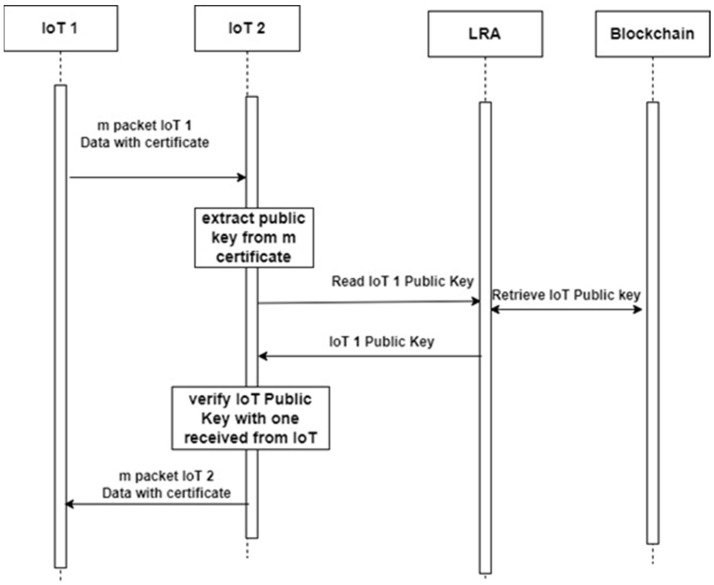
Certificate verification of messages exchanged between IoT devices.

To address this issue, we propose a blockchain-based optimization, illustrated in Figure 5, in which the public key embedded in the device’s certificate is pre-stored and accessed via the DPK blockchain platform. During signature verification, rather than decrypting the certificate signature using the CA’s public key, the verifier retrieves the certificate’s corresponding public key from the blockchain and compares it directly to the key included in the message. This bypasses the need for CA-based decryption and significantly reduces the time complexity associated with traditional PKI-based verification.

#### 3.4.2. Certificate Public Key Reconstruction Offline

In the context of implicit certificates, such as those using the L-ECQV scheme, the proposed approach eliminates the need to reconstruct the public key from certificate reconstruction data and the CA’s public key. This operation is both time-consuming and resource-intensive. Instead, once the reconstruction is performed offline, the resulting public key is stored alongside the implicit certificate in the DPK blockchain. The AA’s public key can also be committed to the blockchain to support future verification and reconstruction, enhancing transparency and trust.

In scenarios involving streaming communication, such as the transmission of (m) messages within a bounded time window, the first message includes the sender’s certificate. Upon reception, the verifying device extracts the public key from the certificate and performs a blockchain lookup to obtain the trusted public key previously stored in DPK. If the keys match, the device caches the validated public key in a local device registry. (Alternatively, even the comparison is not needed as the DPK storage is considered trusted and verified.) For the remaining (m − 1) messages, the receiver skips the blockchain lookup and instead uses the cached public key to perform fast, local signature verification, thereby minimizing cryptographic overhead and communication latency. This certificate verification method is certificate-type agnostic and is compatible with all major certificate formats, including:

- Implicit and explicit L-ECQV certificates
- Standard X.509 certificates
- Self-signed LightCert4IoT certificates
- Any other blockchain-registered certificate format

By decentralizing public key validation and leveraging immutable blockchain storage, the proposed solution ensures scalability, low-latency authentication, and enhanced security across heterogeneous IoT and V2V environments.

## 4. V2V Message Certificate Verification in V2X

**Figure 6:**
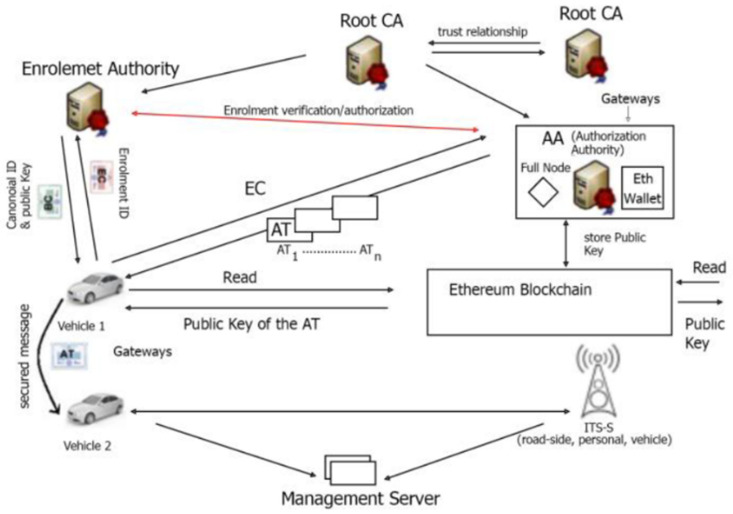
The modification of the V2X standard system by storing the AA public key in the DPK Ethereum blockchain.

To demonstrate the effectiveness of the proposed certificate verification method, we focus on its application within V2V messages without decryption, specifically in V2X environments. These environments consist of highly dense and dynamic networks, making them an ideal testing ground for assessing the method’s robustness and scalability under heavy network traffic and frequent message exchanges. The solution involves a modification related to standardized Vehicle-to-Vehicle (V2V) message signing and verification in Vehicle-to-Everything (V2X) systems. It includes storing the AA’s public keys in the DPK. The message recipient then decrypts the sender’s signature by retrieving the appropriate AA’s public key from the blockchain, linked to the received AT. The key update is that the AA should interact with the blockchain and store its public key there (see the system architecture illustrated in Figure 6) as described in the paper [19].

**Figure 7:**
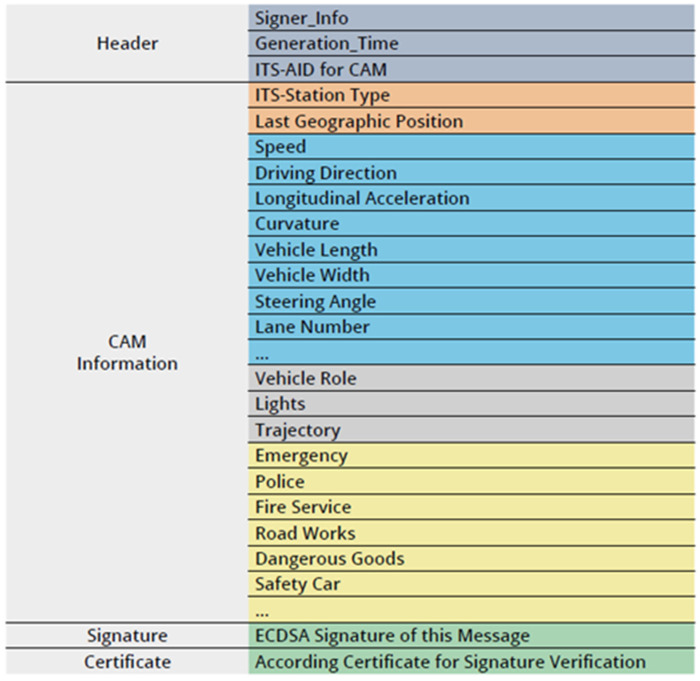
The structure of CAMs [25].

Within the Security Credential Management System (SCMS) framework for V2X communications, transmitted data packets are referred to as Secure Protocol Data Units (SPDUs). Each SPDU comprises three fundamental components: the To-Be-Signed Data (TBSD), a Signer Identifier, and a digital Signature. The TBSD consists of a Header Information field that encapsulates metadata, such as the Generation Time timestamp, and a Signed Data Payload that contains Intelligent Transportation System (ITS) messages, most notably Cooperative Awareness Messages (CAMs), as illustrated in Figure 7. CAMs enable vehicles to disseminate real-time information about their position, velocity, and heading, with transmission intervals regulated by standardized criteria, typically ranging from 10 to 100 ms [21].

The Signer Identifier element contains either an L-ECQV certificate or, as specified in [16], a certificate digest. A critical privacy concern arises if a vehicle persistently uses a single pseudonym certificate over its operational lifespan; adversaries can correlate CAM transmissions across different locations, facilitating vehicle tracking. To address this issue, vehicles use several pseudonym certificates that cannot be linked cryptographically, and they switch between them at regular intervals. This pseudonym change strategy significantly impedes attackers from linking messages and reconstructing vehicle movement trajectories [25].

### 4.1. The Implicit L-ECQV Pseudonym Certificate

**Figure 8:**
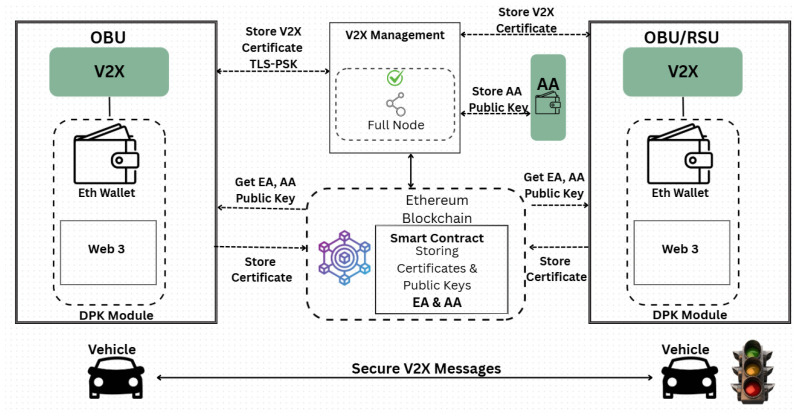
Overview of the V2X system solution based on DPK for the storage of the EA certificate and the storing/reading AA public, pre-storing/reading AT public key from DPK.

The proposed solution consists of a pseudonym L-ECQV certificate called AT, which precomputes the vehicle’s AT public key. This key, along with the associated certificate, is securely stored on a blockchain platform. Upon receiving a message, the vehicle’s receiver queries the blockchain to retrieve the certificate’s precomputed public key, thereby eliminating the computational overhead of reconstructing the public key during each signature verification. AA’s public keys are likewise pre-registered on the DPK blockchain platform as described in [19]. Consequently, when multiple messages (m) with the same certificate are received from a single sender, signature verification leverages the locally cached public key, thereby optimizing processing time. This approach enhances the efficiency and responsiveness of V2V safety-critical message exchanges by signing CAM and DENMs with implicit L-ECQV pseudonym certificates. The system implementation, as illustrated in Figure 8, consists of the following:

- Storing the pseudonym vehicles’ certificates and their precomputed public keys in DPK. The pseudonym certificate public keys are calculated offline once and are not part of the message exchange.
- Storing the AA’s public key.

Our method proposes a precomputed certificate public key and stores the certificate, along with its associated public key, in the DPK blockchain. Receiver devices retrieve and locally store these public keys, ensuring rapid access during signature verification. This approach effectively per-message and improves verification throughput, making it well-suited for resource-constrained IoT and V2X devices.

### 4.2. Certificate Digest in V2V Messages

The certificate digest concept, introduced in [16], addresses the inefficiency caused by transmitting large certificate data alongside each message. Traditional packet exchanges include the full certificate, signature, and payload, leading to significant transmission overhead. The certificate digest is a cryptographic hash of the complete certificate applicable to both implicit and explicit L-ECQV certificates. By storing the certificate’s public key on the receiver device, the need to reconstruct it using reconstruction parameters and the AA’s public key on every verification is obviated. This method reduces packet size substantially by replacing the full certificate with a fixed-size digest and minimizes redundant certificate authenticity verifications by the AA. Under this approach, only the certificate digest is transmitted per packet in V2V communication, resulting in decreased bandwidth consumption and reduced processing latency.

#### Storing the Certificate Digest Public Key in DPK

We align the method described in this paper for certificate digest, which involves storing the certificate digest and its associated public key offline in the DPK blockchain. In that case, receiver devices retrieve and locally store these public keys, ensuring rapid access during signature verification. This approach effectively shortens the certificate data length transmitted per message and improves verification throughput, rendering it well-suited for resource-constrained IoT and V2X devices.

### 4.3. Stream of m Packets Received in V2X Nearby Devices

To accommodate the storage of certificate digests and corresponding public keys for nearby devices, a streamlined data structure is employed. Rather than caching full certificates, the system maintains a compact table containing only the certificate digest (HashedID, H bytes) and the public key (p bytes), resulting in a per-entry storage size of H + p bytes. During high-density scenarios involving N proximate vehicles, the total storage requirement scales to N × (H + p) bytes. To ensure memory efficiency, this data table is periodically refreshed (e.g., every five minutes), purging entries for devices no longer in proximity. Additionally, adopting cryptographic hash functions that produce compact digests further minimizes storage overhead.

### 4.4. System Implementation Sketch

This solution requires adopting the system design and software components described in the paper [26] and adding the pre-registered method for the pseudonym certificate, following the DPK platform based on the Ethereum Blockchain and its API.

Figure 8 illustrates the V2X system solution, along with Sequence Diagrams 8 and 9, which depict the storage of the EA certificate and the retrieval of the AA public key from DPK, respectively.

The solution described in the research papers [19,26] operates on the DPK platform, based on the Ethereum blockchain. This platform provides a versatile foundation for applications that use Ethereum to store application certificates and public keys, as detailed in the Section 2. Figure 8 illustrates the comprehensive solution for the V2X application. The primary components include the DPK platform components described earlier, the PKM software module inside V2X management [13], and the client DPK module. The following mechanisms are part of the DPK platform:

#### 4.4.1. DPK Module

The DPK module acts as the interface between applications and the Ethereum blockchain, mainly for the V2X system. It is installed on both the vehicle’s OBU and the RSU. When the DPK client is set up, it creates an empty Ethereum wallet. During vehicle device setup, this wallet receives Ether from PKM (Wallet Management). This Ether is required to execute smart contract transactions. Performing data storage or modifying a contract’s state on the blockchain requires processing power, which incurs transaction fees paid in Ether. As a result, the vehicle DPK wallet is funded with Ether during configuration and must be registered and installed in the V2X management platform via a web interface or other methods. This platform could be part of the C-ITS or an evolved version of it. The setup involves assigning a unique Token to each DPK client module; the DPK identity itself will be non-addressable. A UUID (Universally Unique Identifier) is used for this purpose, which is common for devices that do not have a directly reachable address, such as behind NATs, including browsers, IoT devices, and mobile devices. This token is crucial for defining and verifying the DPK client module installed on the ITS station device, ensuring the device’s wallet has enough Ether. When the module is downloaded or installed, it generates Ethereum public and private keys and creates an empty wallet. In V2X management, PKM primarily handles wallet management and authenticates the DPK client module within the C-ITS device. The DPK module securely stores the application certificate and public keys in the blockchain. It can be downloaded or embedded into the firmware and can directly access the smart contract via LES or the V2X management server.

#### 4.4.2. The Application Part Includes the Following Components

**V2X application:** This involves C-ITS devices such as OBU and RSU that run V2X software and include a security module. When a user equipment runs the V2X application, it generates and sends the user’s public key or other data to the DPK module, which then transmits this information to the blockchain for secure storage.

**Smart contracts named EA and AA:** Developed on the Ethereum blockchain, they perform key functions such as storing long-term certificates and AA and AT public keys, and retrieving public keys. These contracts handle essential operations for managing user data, including cryptographic public keys.

**The V2X management server:** Part of the C-ITS management framework, this server is hosted on shared servers operated by entities such as governments. It can also run on an MEC server. This server authenticates ITS stations and authorizes requests for certificate issuance and public key storage on the blockchain. A dedicated V2X manager provides access to the blockchain, eliminating reliance on untrusted external providers, such as **Infura.** It may incorporate the certificate authorities EA and AA, follow the standards for managing certificates and public keys in C-ITS, and interface directly with the DPK platform.

**The Authentication and Authorization (AA) node:** This node contains a new software module that connects it with the DPK to store the AA’s public key. The AA node can function as an Ethereum node or connect to the Ethereum network via a light Ethereum node (LES). It creates Ethereum addresses and securely stores its public keys on the blockchain. The node includes a dedicated module (Eth Wallet) for blockchain interactions, communicating through Web 3.0, a wallet, and a predefined API.

#### 4.4.3. Smart Contracts in the Ethereum Blockchain Network: EA+AA

The smart contract serves two primary functions:

1. Use a UUID-indexed data structure to manage and access C-ITS device certificates and public keys. The smart contract stores and retrieves long-term certificates for vehicles and RSUs, along with their associated public keys. No changes have been made to the EA systems or ITSs. Vehicles and RSUs continue to obtain their long-term certificates from EA without modifying the current VPKI, and they manage and read the public keys associated with those certificates within the smart contract.
2. The platform’s smart contract contains the public keys of the AA’s certificate authority nodes, indexed by the AA’s identity and AT’s public key, as outlined in the overall solution. Its main role is to store and retrieve the public keys of both AA and AT.

#### 4.4.4. API Interface to DPK Platform

The API is introduced between the V2X client and the DPK Ethereum platform through the V2X management server or a direct interface to Ethereum via LES. Its principal function is similar to that of other applications on the DPK platform.

*Get the EA and AA public key.*

*Get an EA Certificate.*

*Store the EA long-term Certificate and its public key directly on Ethereum.*

*Store AA public key: The AA public key is stored directly on Ethereum from the AA server.*

*Store the AT public key (after calculation)*

*Read the AT public key*

**Figure 9:**
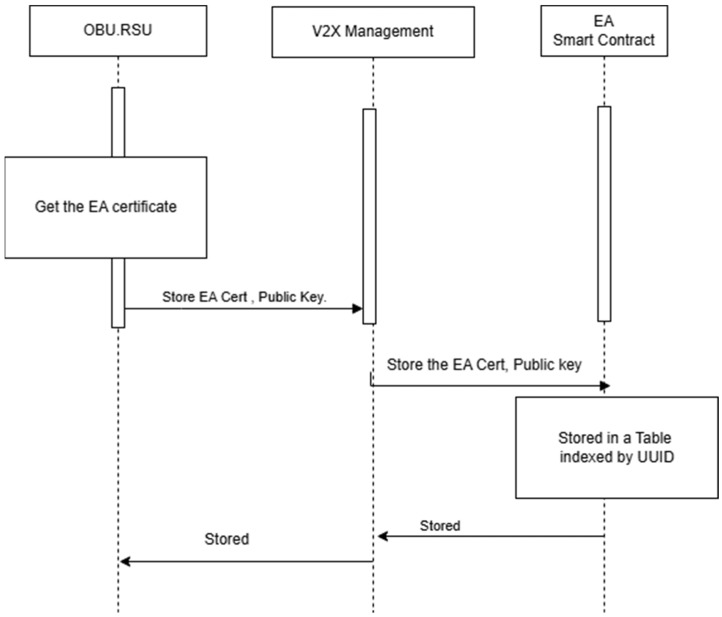
Storage of EA in DPK.

**Figure 10:**
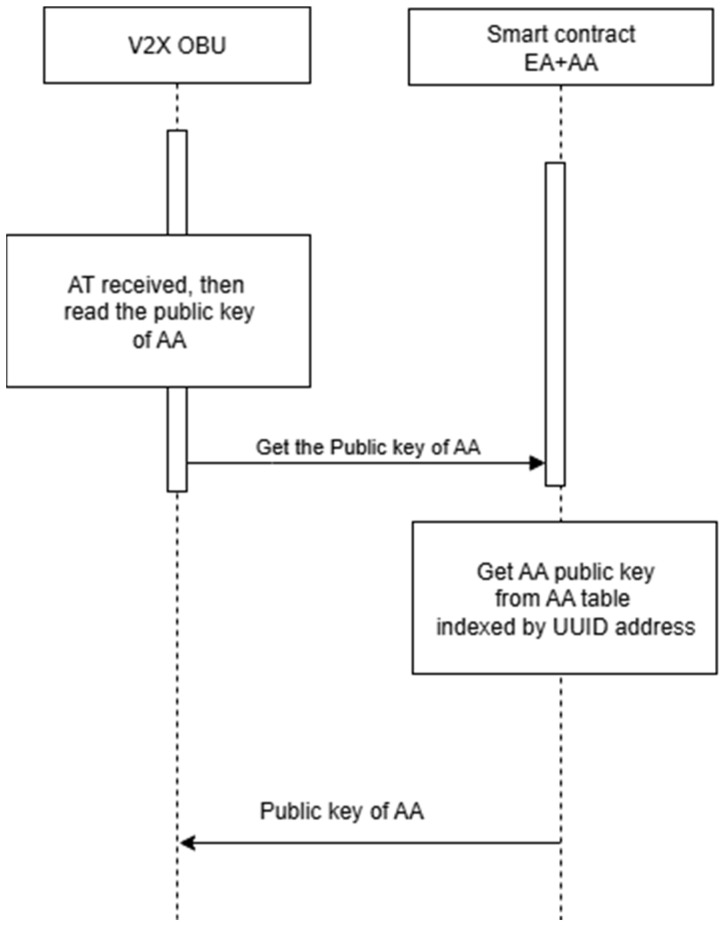
Get the AA Public Key From DPK.

Addressing the V2X Management server by IP and the V2X device by UUID. The V2X server reads the corresponding public key or certificate. Refer to Figure 9, which illustrates the sequence diagram for storing the long-term certificate in DPK. Figure 10 illustrates the sequence diagram for reading AA’s public key.

## 5. Evaluations and Results

### 5.1. Experimental Setup for V2X

The testbed used Ethereum Sepolia’s testnet, along with a Java implementation of the DPK module’s features using Web3j [27]. A Solidity smart contract was deployed on the Ethereum Sepolia test network employing a Proof of Stake (PoS) consensus mechanism [28,29]. Deployment and verification were carried out using Remix IDE, MetaMask, and Etherscan. We used Flask-Web3 [30], an extension that integrates Flask with web3.py, enabling the creation of a Flask app that interacts with an Ethereum client. The smart contract manages mappings between V2X device UUIDs and their associated public keys or certificates, forming a Decentralized Public Key (DPK) Infrastructure for V2X entities. In summary, the initial prototype employs the following technologies:

(1) The Ethereum Sepolia test network provides a development and testing environment.
(2) Solidity smart contracts are used to securely register and manage long-term certificates, public keys, and the AA, AT public key on the blockchain.
(3) The Web3j library allows seamless interaction with the Ethereum network from resource-limited devices.
(4) We utilized a local provider to access the Ethereum blockchain, an HTTP server developed in Flask, which replaced **Infura** for efficient key retrieval. This was enhanced by an in-memory cache, which minimized access times. We built a local cache server with Flask that stores a copy of the blockchain-verified UUID-public key mappings in memory.

We conducted over 100 measurements on the Ethereum 2.0 network, leveraging its Proof-of-Stake (PoS) consensus to improve scalability and reduce transaction costs. Our goal is to measure the following performance aspects:

1. Measure the time to extract and get the public key
2. The time used for storing EA certificates and AA, AT public keys in the Ethereum network.
3. The storage cost of a public key or certificate.

#### 5.1.1. The Time Needed to Retrieve the EA Certificate and AT, AA Public Key in the DPK Platform

**Figure 11:**
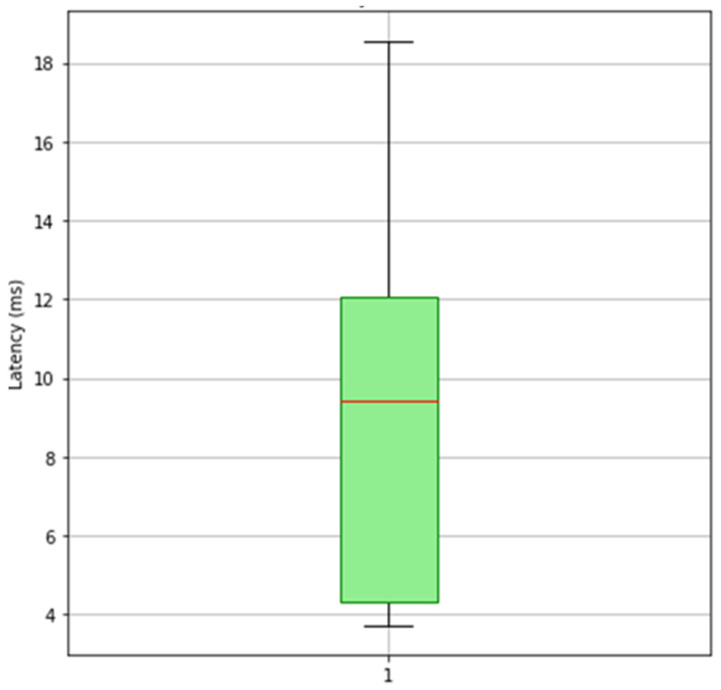
Retrieval Public keys Box plot.

**Figure 12:**
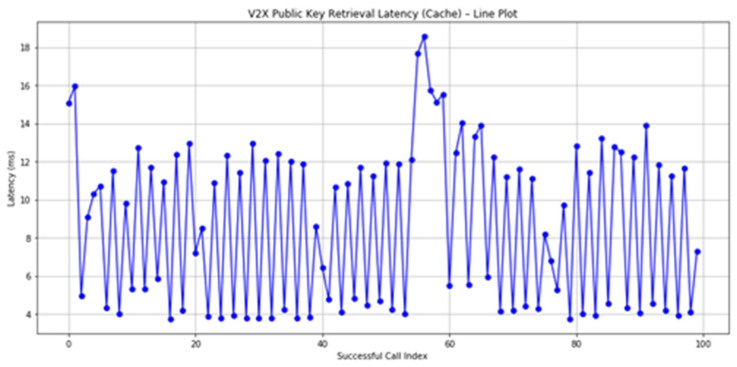
Retrieval of 100 Public keys diagram.

Our measurements indicated an average retrieval time of approximately **8.7176** ms across 100 calls for public keys. As shown in Figure 11 and Figure 12, retrieving public keys is well within the required speed for real-time authentication. This duration is influenced by network latency and the speed of Remote Procedure Calls (RPCs).

#### 5.1.2. The Storage Cost of a Public Key or Certificate

The cost is approximately 115,854 GAS; 0.00030399 ETH (~0.77 USD), demonstrating the economic viability of DPK on a PoS chain for large-scale V2X deployments.

#### 5.1.3. The Time Used for Storing EA Certificates and AT, AA Public Keys in the DPK Platform

The average time to complete a certificate or public key storage transaction was about 3891.75 ms.

### 5.2. Evaluation and Analysis of the Test Results of a Stream of m Messages and V2V

Since there are minimal differences in key generation and signature generation, this subsection primarily focuses on signature verification. In the L-ECQV certificate method, each verification requires combining the public key reconstruction data from the certificate with the public key from the certificate authority to derive the actual public key. This process often takes considerable time due to the complexity of EC point calculations, resulting in longer verification times for the L-ECQV certificate method.

In our proposal, the AA’s public keys are stored in DPK, and the AT certificate public keys are calculated once and prestored, enabling a one-time setup.

Empirical performance data from [16] shows that when using the NIST P-256 elliptic curve:

- The median time for signature verification using a lightweight explicit certificate is approximately 4.67 ms.
- For a lightweight implicit certificate, the verification time increases to 44.47 ms due to the additional reconstruction step.
- The certificate digest method achieves a verification time of 4.91 ms.
- Signature verification of the proposed blockchain-integrated model:
- The calculation of the certificate public key is done once offline.
- Signature verification is **conducted once per communication stream and usually takes around 8.7 ms, independent of encryption strength**. This time includes getting the public key of the certificate, which was pre-calculated and stored offline in the DPK.

In conclusion, verifying signature messages in V2V typically takes around 8 ms, regardless of the number of messages (m) carrying the same certificate from the same source vehicle. This performance makes the method especially well-suited for applications where certificates are reused across multiple messages, such as in Vehicular Ad Hoc Networks (VANETs) [31] or real-time sensor networks. The proposed solution offers lower latency than the existing system, as shown in Section 5.3.

The proposed method for V2V messages, which utilizes the implicit L-ECQV or certificate digest, achieves a shorter certificate length while maintaining higher verification efficiency. Therefore, it is suitable for the transmission and computational environments in dense IoT devices and V2V messages. However, since it requires temporary storage for the certificate and public key values, it will necessitate some storage space on the IoT device, which is not an issue for V2V. The amount of storage required will depend on the specific application context of the IoT device. This issue can be addressed by adjusting the value of received messages from the same source (m) mentioned, according to the application requirements. Furthermore, different values of (m) have been analyzed in [16], with the results indicating that the proposed method can be effectively applied.

This optimization yields considerable performance benefits in high-density IoT environments, such as scenarios involving 10,000 co-located devices. In such cases, each public key is read from the DPK blockchain only once, eliminating the need to perform computationally expensive signature verifications for every received packet. This dramatically reduces the overall cryptographic overhead. It is important to emphasize that the effectiveness of this approach relies on the pre-storage of public keys on the blockchain platform. If the public key is not already available, the corresponding certificate must be transmitted and authenticated through conventional means. Nevertheless, the total memory requirement for storing all necessary public keys remains below 1 Mbyte, based on experimental analysis reported in [16]. This is well within the storage constraints of most modern IoT devices.

### 5.3. V2V Latency in the Existing Systems

The time required for a receiver to process a CAM or DENM in V2V communication depends on factors such as the communication technology (ITS-G5 or LTE-V2X), message size, and the receiver’s processing setup. The total message handling time consists of:

- Transmission Delay, which is the time it takes to send the message wirelessly (usually 1–10 ms for ITS-G5 or LTE-V2X);
- Reception and Decoding, the time needed to receive and interpret the message.

#### 5.3.1. CAMs

A study conducted on the Smart Highway testbed in Antwerp utilized timestamping to measure CAM latency [32].

- ITS-G5 (IEEE 802.11p [33]): Average latency for regular CAMs was around 10–20 ms.
- LTE-V2X: Slightly lower latency, often under 10 ms, due to semi-persistent scheduling and better channel access control

#### 5.3.2. DENMs

According to ETSI TS 102 637-3 V1.1.1 [34].

- ITS-G5 (IEEE 802.11p): The average DENM latency is approximately 15–25 ms, which is slightly higher than that of CAMs due to additional processing.
- LTE-V2X: Usually under 15 ms, thanks to semi-persistent scheduling and efficient resource management

Transmitting a message that implements the V2V application according to the ETSI specification [34] should not exceed 20 ms.

Table 5 summarizes the proposal features, and Table 6 compares the existing and blockchain signature verification.

### 5.4. Evaluation of the Handshake Time Protocols Based on the Signature Verification on the Blockchain

We base our evaluation on the experiments reported in [12] for the DTLS protocol, which are similar to those of other secure protocols during the handshake phase. The testing results performed for the DTLS protocol are presented in [12]. Establishing a secure DTLS handshake using certificate-based authentication is a computationally intensive process, primarily due to the complexity of validating certificate chains and the associated cryptographic operations, and the results showed the following:

In existing DTLS, the cryptographic overhead per handshake, even with a minimal chain of two certificates, can reach 15 s, posing significant constraints on latency-sensitive or real-time applications. Additionally, a full DTLS handshake consists of up to 15 protocol messages, many of which may need to be fragmented to comply with the MTU (Maximum Transmission Unit) constraints at the DTLS or lower layers. Even relatively short certificates often exceed 220 bytes, surpassing the frame-size limits of typical link-layer technologies (e.g., IEEE 802.15.4, BLE) [35], thereby increasing the number of transmitted packets and further exacerbating handshake latency. These performance challenges underscore the need for optimized certificate management and lightweight cryptographic alternatives in IoT and constrained networking environments.

The blockchain DTLS indicates that fetching the public key from the blockchain is effectively instantaneous **(latency ≤ 5 ms)** and incurs no direct retrieval cost, as the mapping data is publicly accessible. This demonstrates the practical feasibility of the blockchain-enabled handshake. These numbers can be validated or extrapolated to other similar protocols, such as TLS and IKEv2.

Performance analysis of X509 PKI/CA vs. a blockchain-based solution, using LightCert4IoT as an example. A comparison of DTLS and the new blockchain-based method is presented in Table 7.

## 6. Conclusions

This paper presents an innovative certificate verification mechanism that integrates blockchain technology—specifically certificates with pre-stored public keys—into secure communication protocols such as DTLS and TLS. This integration significantly reduces handshake duration and mutual message authentication between IoT devices, with a focus on vehicle-to-vehicle (V2V) messaging in dense IoT environments. The core novelty lies in offloading certificate verification to a decentralized blockchain, thereby eliminating the need to repeatedly transmit full certificates and reducing the computational burden of traditional public key infrastructure (PKI) during the handshake phase. By leveraging implicit L-ECQV certificates and certificate digests stored on a distributed public key (DPK) blockchain, the proposed method enables vehicles and IoT devices to perform rapid, one-time signature verification with negligible latency in V2V messaging, especially under high-load scenarios such as V2X communications.

Empirical evaluations conducted on the Ethereum Sepolia testnet demonstrate that public key retrieval via the blockchain is nearly instantaneous (≤8 ms), in contrast to the 15 s average delay observed in traditional certificate-based DTLS handshakes involving full X.509 chains. Furthermore, the proposed solution achieves substantial packet size reduction and efficient storage utilization—critical benefits for resource-constrained IoT devices operating in real-time environments.

This research aims to further reduce latency in V2V communication by building on the DPK architecture described in Section 2. This involves replacing the decryption of the AT certificate signature with verification of the AT public key, which is pre-stored in the DPK blockchain. The proposed solution significantly reduces cryptographic overhead in V2V mutual authentication for V2X communications, achieving a maximum latency of just 5 ms. It is performed once per message stream for a specific AT and produces message subsets, enhancing performance in high-density deployments with over 10,000 devices. The public key embedded in the device’s certificate is pre-stored and retrieved from the DPK blockchain platform. During signature verification, instead of decrypting the certificate signature with the AA’s public key, the verifier fetches the corresponding public key directly from a trusted blockchain platform. This approach eliminates the need for AA-based decryption and significantly reduces the time complexity of traditional PKI-based verification.

For future work, the solution outlined in this paper can be adapted for private blockchains in industrial environments, allowing only authorized users to access network data through a custom consensus algorithm that boosts performance. However, this will require developing a new implementation paper that extends the proof-of-concept on Ethereum to a private blockchain. Additionally, large-scale testing in real-world V2X scenarios will be performed to assess performance with emerging vehicular communication standards, including 5G NR V2X [36] and IEEE 1609.2.

## 7. Related Work

Several academic works have attempted to leverage blockchains for decentralized trust management in Connected and Automated Transportation Systems (C-ITS) frameworks. Replacing the centralized PKI/CA, however, none of these papers tackled the efficiency of signature verification of IoT and V2X via blockchain integration. We list below some papers related to blockchain as an alternative to the PKI/CA.

Benarous et al. introduce a blockchain system for pseudonym management, enabling vehicles to generate their own pseudonyms without requiring oversight from authorities. The framework operates with two main blockchains: one for storing pseudonym certificates and verifying their validity, and another for handling revocations [37].

Hui et al. [38] introduce a comprehensive access control scheme for VANET data using blockchain technology (FADB). This approach combines ciphertext-based attribute encryption (CP-ABE) with the Ethereum blockchain and the Interplanetary File System (IPFS) to support distributed storage with detailed access controls. In this system, the blockchain eliminates the need for a centralized PKI, simplifying user identity management and data storage.

Zhuo et al. [39] introduced a decentralized key management system for C-ITS called DB-KMM, which uses blockchain and smart contracts to automate the registration, updating, and revocation of public keys for various stations. They also proposed an innovative protocol for mutual authentication and key exchange. The system employs smart contracts to oversee key updates and revocations across different users.

Yang et al. [40] proposed a decentralized trust management system based on blockchain for C-ITS environments. Their method enables ITSS-Vs to verify messages from nearby vehicles using the Bayesian Inference Model. ITSS-Rs evaluate trust by using ratings uploaded by ITSS-Vs, adjusting trust value offsets for involved vehicles, and compressing this data into a block. Each ITSS-R then attempts to add its block to a shared trust blockchain used by all ITSS-Rs. Similarly, Lei et al. [41] employed blockchain technology to facilitate key transfers between security managers within ITS communications.

Chulerttiyawong et al. leverage consortium blockchain technology to issue pseudonym certificates across multiple jurisdictions in a road network. Their approach uses smart contracts to issue vehicle certificates, replacing the traditional centralized PKI with a decentralized blockchain system. Additionally, the blockchain and its smart contracts handle the revocation of pseudonym certificates [42].

## Figures and Tables

**Table 1:** The comparison of elliptic curve point lengths (units: bytes).

EC	Uncompressed EC Point	Compressed EC Point
NIST P-192	49	25
NIST P-224	57	29
NIST P-256	65	33
NIST P-384	97	49
NIST P-521	133	67
Brainpool P-192	49	25
Brainpool P-224	57	29
Brainpool P-256	65	33
Brainpool P-384	97	49
Brainpool P-512	129	65

**Table 2:** Structure of a lightweight implicit certificate L-ECQV.

Field	Byte(s)	Description
Type	1	Type
Serial Number	8	Octet String—Unique Serial Number
Curve	1	EC Choice
Hash	1	Hash Algorithm Choice
Issuer ID	8	Octet String—Identifier of CA
Valid from	5	Beginning of Validity in Unix Time
Valid to	4	End of Certificate Validity
Subject ID	8	The subject ID identified by Organizationally unique identifier (EUI-64)
Usage	1	Digital Signature (0x00)
**Public Key**	**P**	**The public key reconstruction data (i.e., an EC point)**

**Table 3:** The comparison of ECDSA signatures’ lengths (units: bytes).

EC	Signature with Uncompressed EC Point	Signature with Compressed EC Point
NIST P-192	73	49
NIST P-224	85	57
NIST P-256	97	65
NIST P-384	145	97
NIST P-521	199	133
Brainpool P-192	73	49
Brainpool P-224	85	57
Brainpool P-256	97	65
Brainpool P-384	145	97
Brainpool P-512	193	129

**Table 4:** Structure of explicit L-ECQV Certificate.

Field	Byte(s)	Description
Type	1	Type
Serial Number	8	Octet String—Unique Serial Number
Curve	1	EC Choice
Hash	1	Hash Algorithm Choice
Issuer ID	8	Octet String—Identifier of CA
Valid from	5	Beginning of Validity in Unix Time
Valid to	4	End of Certificate Validity
Subject ID	8	The subject ID identified by Organizationally unique identifier (EUI-64)
Usage	1	Digital Signature (0x00)
Public Key	P	The public key reconstruction data (i.e., an EC point)
**Signature**	**S**	**An ECDSA Signature**

**Table 5:** Summary of the proposal’s Features.

The calculation of the L-ECQV and the digest certificate public key	The process is completed offline, and the public key certificate is stored in DPK. This applies to all types of certificates and the storage of the CA public key.
Signature verification	Executed only once per communication stream and consistently based on retrieving the certificate public key stored in DPK within an average retrieval time of 8.7 ms.
Method applicability	The proposed method is suitable for the transmission and computational environments in dense IoT devices and V2V messages.
Latency in the case of V2V and pseudonym certificate AT	Suppose the AT’s valid duration is around 10 min. Instead of decrypting the signature AT for every message, it is decrypted just once, reducing the total verification time of ATs to about 8 ms.
Performance	In high-density IoT environments, such as scenarios with 10,000 co-located devices, each public key is read from the blockchain only once, eliminating the need to perform computationally intensive signature verification for every received packet.

**Table 6:** Comparison between existing and blockchain signature verification.

The median time for signature verification of an explicit certificate	Approximately 4.67 ms.
The median time for signature verification of an implicit certificate	44.47 ms due to the additional reconstruction step.
The median time for signature verification for the certificate digest	verification time of 4.91 ms
certificate verification based on blockchain	
Signature verification of the proposed blockchain-integrated model.	The certificate public key is calculated offline and stored on the blockchain, and it applies to all types of certificates. The storage of the CA public key on the blockchain Signature verification is performed **only once per communication stream** and is consistently based on retrieving the certificate public key stored on the blockchain, with an average retrieval time of **8.7 ms**.
The time required to retrieve the EA certificate and the AT, AA public keys on the DPK platform.	The average retrieval time of approximately **8.7176** ms across 100 calls for public keys.
The storage cost of a public key or certificate in DPK.	The cost is approximately 115,854 GAS; 0.00030399 ETH (~0.77 USD).
The duration for storing EA certificates and AT, AA public keys on the DPK platform.	The average time to complete a certificate or public key storage transaction was about 3891.75 ms.

**Table 7:** DTLS Performance based on X509 vs. LightCert4IoT based on Blockchain.

Metric	Based on X509 PKI/CA	LightCert4IoT Blockchain
CoAP/DTLS Handshake		
Gain in processing times	12s	<6 s
Transmission Overheads	Packet fragmentation can increase the total number of transmitted packets when certificates exceed 200 bytes.	No packet fragmentation
Interface to Ethereum Retrieving Data: Public Key	Not applicable	<5 ms

## Data Availability

The original contributions presented in this study are included in the article. Further inquiries can be directed to the corresponding author.
